# Electrical Field Stimulation with a Novel Platform: Effect on Cardiomyocyte Gene Expression but not on Orientation

**Published:** 2012-06

**Authors:** Kirsi Kujala, Antti Ahola, Mari Pekkanen-Mattila, Liisa Ikonen, Erja Kerkelä, Jari Hyttinen, Katriina Aalto-Setälä

**Affiliations:** 1 *Institute of Biomedical Technology, University of Tampere, FIN-33014 University of Tampere, Tampere, Finland;*; 2 *BioMediTech, Tampere, Finland;*; 3 *Department of Biomedical Engineering, Tampere University of Technology, P.O. Box 692, FIN-33101 Tampere, Finland;*; 4 *Finnish Red Cross, Blood Service, Helsinki, Finland;*; 5 *Heart Center, Tampere University Hospital, Teiskontie 35, 33520 Tampere, Finland*

**Keywords:** cardiomyocytes, collagen gel, connexin-43, electrical stimulation, orientation, beta myosin heavy chain 7, gene expression

## Abstract

Electrical field stimulation has been shown to improve cardiac cell alignment and functional properties. In this study, neonatal rat cardiomyocytes were exposed to both long-term and short-term stimulation with the goal of investigating whether it is possible to achieve cell orientation and the maturation of cardiomyocytes with a novel, microelectrode array (MEA)-compatible electrical stimulation platform. Cells were viable after electrical stimulation, but no orientation or other morphological changes were observed. However, the electrode wires in MEA dishes affected the cell orientation. Cell contractions synchronized with pacing, but settled back to their original frequency in the absence of stimulation. The expression of genes encoding a gap junction protein connexin-43 (Cx-43), and contractile cardiac protein beta myosin heavy chain 7, was stronger in stimulated cells than in controls (*p*<0.05). In summary, the surface topography influenced to cardiomyocyte orientation, suggesting that the micro architecture of the biomaterials should be carefully designed for cell applications. However, as electrical stimulation and its duration affected gene expression of some main cardiac proteins, the stimulation system may prove useful to enhance the cardiac differentiation of stem cells.

## INTRODUCTION

The ultimate goal in cardiac tissue engineering is to generate heart muscle with the morphological and functional properties of natural myocardium. Pluripotent human embryonic stem cells (hESC) and human induced pluripotent stem cells (hiPS) can differentiate into cardiomyocytes (CM) (14, 34), and with the help of stem cell technology, it could be possible to generate a tissue model for cardiac studies and eventually to create a graft to replace damaged heart tissue. However, one major problem in culturing CMs has been the lack of cell orientation and immature phenotype.

Contractile and electrophysiological functionality as well as cell alignment, mechanical stability and the expression of cardiac cell markers are among the most important characteristics of engineered cardiac constructs. Electrical field stimulation has proposed to improve cell differentiation, alignment and functional properties, and is therefore considered a crucial parameter for the generation of synchronously contracting cardiac cells. Even though electrical field stimulation studies have been carried out mostly on neonatal rat CMs (NRCs), electrical fields have also served in the cardiac differentiation of mouse embryonic stem cells (27) and hESCs (28). Electrical stimulation has been shown to induce cell elongation (3-5, 9, 24) and alignment (3, 11, 24), both affecting cell functionality. In addition, reports indicate that electrically stimulated CMs have a preferred orientation in response to field stimulation. Namely, it has been demonstrated that CMs are more excitable when the long axis of the cell is oriented parallel to the electrical field (30). Also, a marked level of ultra structural organization has been observed due to stimulation by means of centrally positioned elongated nuclei and well-aligned registers of sarcomeres (24). As for functional properties, stimulation has induced regular excitation-contraction coupling between electrical pacing signals and macroscopic contractions, which is necessary for the development of contractile behavior (9, 24). In addition, stimulation has induced other functional properties such as low excitation threshold (9), amplitude of synchronous contractions (24), stabilization of action potential duration (26) and an increase in the calcium current peak (6). Electrical stimulation has also led to enhanced calcium transients (12) and the development of more organized myofibrils (20), while stimulated CMs have demonstrated well-developed contractile apparatuses (3, 24). On the molecular level, electrical stimulation has induced a rise in myosin heavy chain (MHC) levels (24), which correlates with the contractile velocity of cardiac muscle (22). Electrical stimulation has also increased the production of gap junction protein Connexin-43 (Cx-43) (4, 5, 9, 24) as well as expression of the cardiac genes of myosin light chain-2 and atrial natriuretic factor (20).

Electrical stimulation has been studied with various stimulation systems using current- or voltage-based stimulation. Optimization of the stimulation parameters is based on those found in both the developing and the native heart (i.e., in the hearts of one-week-old rats) (3, 9). The frequency of 1 Hz has been used because it is physiological for humans and at the low end of the physiological regime for rats (3). In earlier studies, rat CMs have typically been stimulated with a field strength of around 5 V/cm with monophasic or biphasic waveforms (3, 9, 12, 24).

The main objective of this study was to investigate whether electrical field stimulation with our novel platform could enhance CM orientation and maturation as well as improve functional properties. NRCs were cultured as monolayers on collagen gel or gelatin-coated micro electrode array (MEA) chambers and stimulated. The cell viability, electrophysiological and morphological properties, as well as protein and gene expression of cardiac cell markers were analyzed to determine the effect of this novel device on functional and molecular properties of NRCs.

## MATERIALS AND METHODS

### Cell culture

NRCs were chosen to this study because they are easy to obtain in large amounts and they provide a suited cell model of beating CMs. NRCs were harvested from the whole-hearts of two- to five-day-old rats as described earlier (31). The hearts were removed and enzymatically dissociated with collagenase type II solution and seeded as cell density of 282 000 cells/cm^2^ onto collagen gel or 0.1% gelatin-coated MEA chambers in Culture Medium I [CMI, Dulbecco’s Modified Eagle’s Medium/Ham’s Nutrient Mixture F12 (DMEM/F-12, Sigma-Aldrich, Germany), 10% Fetal bovine serum (FBS, Gibco, Finland), 100 IU/ml Penicillin/ 0.1 mg/ml Streptomycin (P/S, Gambrex, Belgium), 2.56 mM L-glutamine (Sigma-Aldrich, Germany)]. After first day and thereafter cells were precultured in 1 ml of Complete Serum Free Medium [CSFM, DMEM/F-12, 10% Bovine serum albumin (BSA, Sigma-Aldrich, Germany), 2.8 mM Sodium Pyruvate (Cambrex, Belgium), 2.56 mM L-glutamine, Insulin-transferrin-sodium selenite media supplement (ITS, Cambrex, Belgium; 1 µM insulin, 5.64 µg/ml transferring, 32 nM selenium), 100 IU/ml P/ 0.1 mg/ml S, 0.1 nM 3,3’,5-Triiodo-L-thyronine sodium salt (T3, Sigma-Aldrich, Germany)] in a 37°C/5% CO_2_ to allow the cells to attach to MEA chambers. Culture medium was changed every or every second day. Cells serving as controls were cultured in the same way as electrically stimulated cells were. The animals were sacrificed according to guidelines of the Animal Unit, Medical School, University of Tampere and the Ethical Committee of the Animal Unit has accepted the method to obtain the cells.

### Collagen gel

The collagen was isolated from adult Sprague Dawley rat tails (10) and stored at -20°C. In brief, tails were thawed and cut into pieces, the skin was then removed, and the collagen fibers were pulled out and placed into 0.1% acetic acid. The fibers were dissolved into acetic acid, and the collagen was precipitated with 25 v-% sodium chloride. The solution was then centrifuged, and the collagen pellet was again dissolved in 0.1% acetic acid. To balance the salinity, the solution was dialyzed at 4°C. Finally, the concentration of the collagen was determined with a Pierce^®^ BCA Protein Assay kit (Thermo Scientific, USA) using BSA as the standard. The concentration was 1.043 mg/ml.

The gelation of the collagen (19) was performed as described earlier, with some modifications. At first the collagen solution was placed on ice, and two different neutralizing buffers [12 mg/ml NaHCO_3_ (Sigma-Aldrich, Germany) in 0.1 N NaOH (Merck, Germany) and 1.3 M NaCl (Baker, the Netherlands) in 0.2 M Na_2_HPO_4_ (Baker, the Netherlands)] were added. The solution was then incubated at 37°C for 30 min to allow for gel formation.

### Electrical stimulation setup

**Figure 1:**
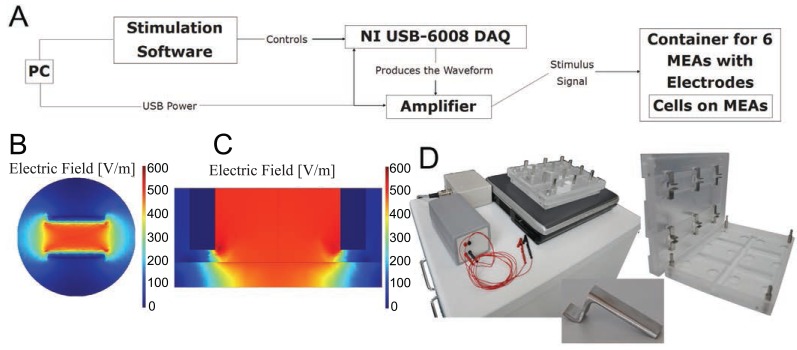
**(A)** The stimulation system. The stimulation sequence is designed with PC software that controls the NI USB-6008 DAQ device. The waveform that the device produces is amplified and delivered to the MEA container, where the cells reside on MEA dishes; **(B, C)** The simulated result of the electrical field on an MEA dish; **(B)** If the cells to be stimulated are placed on the MEA electrodes in the middle of the MEA dish, they will experience a fairly uniform 500 V/m electrical field. The image is plotted at the height of 0.1 mm; **(C)** A lateral view from the center line shows that the electrical field diminishes at the bottom of the MEA close to the electrodes. The MEA dish has been included in the simulation as an insulator at the bottom; **(D)** The designed stimulation system, consisting of the stimulation electronics, PC, and the MEA dish container; the MEA dish container appears in detail on right, with an individual electrode shown in detail at the bottom.

The in-house designed and developed system comprised three parts: electronics for stimulation waveform generation and amplification, an MEA dish container with electrodes, and stimulation software running on a PC laptop (Figure 1A and 1D). The functionality of the system was tested electrically, and its effects on the cells were observed with a microscope.

The electrode holder manufactured from polymethyl methacrylate also worked as a container and was designed to serve as a petri-dish as well. The container was designed to house six MEA dishes. For each MEA dish, two 10-mm-wide stainless steel plate electrodes were placed 5 mm apart to create a homogeneous field for stimulation. The electrode setup and the stimulus field were simulated with the finite element method using Comsol Multiphysics software to assess the homogeneity of the field across the whole cell culture (Figure 1B and 1C). To ensure that the stimulation field is as uniform as possible, the electrodes were set to 0.5 mm from the bottom. Air exchange was enabled via a small space between the container and the lid, which was designed to feature a 10 mm protruding rim to keep the container closed. To counteract evaporation, the bottom part of the container was designed to provide pools for sterile water. The lid and the bottom part featured small glass windows which allow the user to observe the cell cultures on the MEAs with a microscope.

The electronics of the system were designed to yield a field strength of at least 5 V/cm, as similar field strengths had proved effective (23). The electronics consisted of a single-sided supply operational amplifier circuit which amplifies the signal created by a PC-controlled National Instruments USB-6008 Data Acquisition (DAQ) device and produces a voltage-based stimulus. The system was designed to be USB powered. The user can set the stimulation voltage, frequency, total stimulation time, pulse duration, and delays between pulses. The software allows users to design stimulation waveforms, thus enabling them to use uncommon pulse shapes. The system produces electrical fields with a maximum of 5.3 V/cm and an output frequency ranging from 0.5 to 40 Hz. The shortest pulses produced were 2 ms wide. Two clips and wiring connected the electronics to the electrodes. Waveform production using the DAQ device was controlled with the software, which allows the user to adjust the required stimulation parameters and to queue different stimulation sequences.

The electrical output of the stimulator, from both the stimulation electrodes and the MEA electrodes, was verified with an oscilloscope. In addition, the effect of stimulation on medium pH levels was measured with pH electrode (AMANI-1000, Innovative Instruments, Inc, Tampa, USA) from stimulated and control samples after a long-term stimulation with the same parameters used in experiment I (Table 1).

**Table 1:** Experimental groups

	Pulse form	Pulse duration	Electrical field	Frequency	Duration of stimulation	Culture material	n^a^

Experiment I	Biphasic	100 ms / 100 ms	5.3 V/cm	1 Hz	3 d	2D-coating	3/3
Experiment II	Biphasic	2 ms / 2 ms	5.3 V/cm	1 Hz	2 d	2D-coating	3/5
Experiment III	Monophasic	2 ms	5 V/cm	1 Hz	3 d	2D-coating	4/5
Experiment IV	Monophasic	2 ms	5 V/cm	1 Hz	3 d	collagen gel	6/6

a Control/stimulated samples.

### Stimulation protocols

**Pacing experiment.** Delivery of the stimulation pulses to the cells and the effect of stimulation on the cell culture and beating frequency was tested by observing the behavior of NRCs with a microscope (Olympus IX51, Olympus Corporation, Tokyo, Japan). The cells were seeded on gelatin-coated MEA chambers and paced for 20 s with both monophasic and biphasic pulses at 2.5 and 5 V/cm and a frequency of 1, 2, and 3 Hz with pulse durations of 2 ms, 4 ms, and 10 ms.

**Figure 2:**
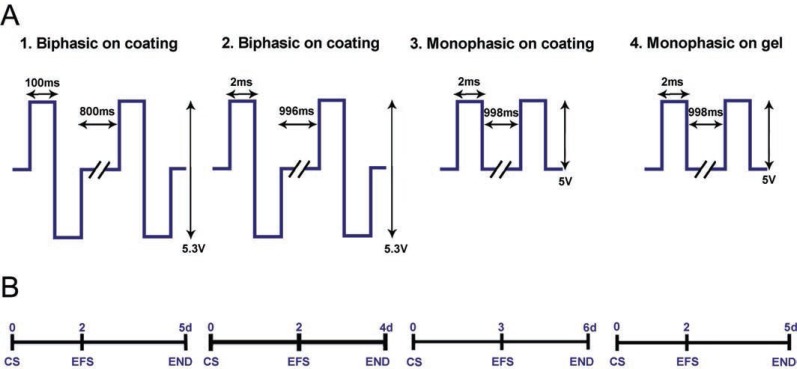
Experimental design. **(A)** Experimental groups for long-term stimulation where NRCs were cultured either on gelatin coating (1-3) or on collagen gel (4). Parameters used for electrical stimulation were biphasic 100-ms square pulses at 5.3 V/cm and 1 Hz (1), biphasic 2-ms square pulses at 5.3 V/cm and 1 Hz (2), and monophasic 2-ms square pulses at 5 V/cm and 1 Hz (3 and 4). **(B)** Experimental timelines for each stimulation group. Control samples were cultured for the same amount of time, but without electrical stimulation. CS, cell seeding; EFS, electrical field stimulation initiation; END, experimental ends.

**Long-term stimulation.** After preculture, MEA chambers were transferred to the stimulating chamber. The cells were cultivated for 48 to 72 h without electrical field stimulation in order to allow the cells to attach; electrical field stimulation followed for an additional 48 to 72 h using either monophasic or biphasic pulses at 5 V/cm or 5.3 V/cm with 2 ms, 4 ms or 200 ms pulse durations and a frequency of 1 Hz (Figure 2). Constructs cultured without electrical stimulation served as controls. There were four independent experimental series and three to six samples in both the stimulation and control groups within each experimental series. Electrical field stimulation stopped on days 4 to 6, and cells were processed either for live/dead staining, immunocytochemistry, or for RNA isolation. More detailed information on each experimental series appears in Table 1.

### Electrophysiology - microelectrode array (MEA)

The electrical activity of beating NRCs was monitored using the microelectrode array (MEA) system (Multi Channel Systems MCS GmbH, Reutlingen, Germany). Measurements were made of cells on gelatin coating at 37°C, and signals were recorded for 2 min via every microelectrode. The sampling frequency was 20 kHz. Signals were measured before and after stimulation from the first, second, and third experimental samples, and in addition, signals from the second experimental samples were measured twice during the stimulation period. Signals from the control samples were measured at the same time points.

### Cell stainings

**Live/dead staining.** CMs were live/dead stained using a LIVE/DEAD^®^ Viability/Cytotoxity kit for mammalian cells (Molecular Probes, Inc., Invitrogen, USA). Staining was carried out according to Molecular Probes’ instructions. 0.1 µM Calcein AM staining live cells green and being seen at wavelength of 488 nm and 0.5 µM Ethidium homodimer-1 staining dead cells red and being seen at wavelength of 568 nm were added to the culture medium and incubated at room temperature. The labeled cells were viewed and photographed with a Nikon Eclipse TE2000-S phase contrast microscope with fluorescence optics and a Nikon COOLPIX 5400 camera.

**Immunocytochemistry.** The cells were fixed in MEA chambers with 4% PFA in phosphate-buffered saline (PBS) (0.01 M, pH7.4) for 20 min at room temperature (RT), followed by washing with PBS (2 × 5 min). The cells were then permeabilized and blocked with 0.1% Triton X-100, 1% BSA (Sigma-Aldrich, Germany), and 10% normal donkey serum (Sigma-Aldrich, Germany) in PBS for 45 min at RT. Primary antibodies anti-troponin I (Santa Cruz, USA) (1:500) and anti-cardiac troponin T (Abcam, UK) (1:1500) were incubated overnight at 4°C. Alexa Fluor 568-conjugated donkey anti-goat served as a secondary antibody (1:800) (Invitrogen, USA) and finally the cells were mounted with Vectashield (Vector Laboratories, USA) containing 4’,6-diamidino-2-phenylindole (DAPI) for staining nuclei.

### RT-PCR

Total RNA was isolated with a NucleoSpin^®^ RNA II kit (Macherey-Nagel GmbH & Co., Germany). The concentration and quality of the RNA was monitored spectroscopically (Nanodrop, Wilmington, DE, USA) and 100 ng of total RNA was transcribed to cDNA in a total volume of 20 μl with a High Capacity cDNA Reverse Transcription kit (Applied Biosystems, USA). The PCR reaction consisted of 1 μl cDNA, 24 μl of 2 × PCR Mastermix (Applied Biosystems, USA), and 200 nM of each primer. The expressions of three cardiac markers were evaluated: alpha myosin heavy chain 6 (MYH-6), beta myosin heavy chain 7 (MYH-7), and connexin 43 (Cx-43). GAPDH served as a housekeeping gene and calibrator of all cells (Primers: Table 2).

**Table 2:** Primer sequences

Gene	Forward Primer	Reverse Primer	Size (bp)

RT-PCR			
GAPDH^a^	TGGAAAGCTGTGGCGTGATG	TCCACCACCCTGTTGCTGTAGC	380
MYH6^a^ (alpha myosin heavy chain 6)	GGAAGAGCGAGCGGCGCATCAAGG	CTGCTGGACAGGTTATTCCTCA	304
MYH7^a^ (beta myosin heavy chain 7)	GCCAACACCAACCTGTCCAAGTTC	TCAAAGGCTCCAGGTCTCAGGGC	201
Cx-43^a^ (connexin 43)	CATTGGGGGGAAGGCGTGAGG	AGCGCACGTGAGAGATGGGGAAG	401
Q-PCR			
β-actin^b^ (beta actin)	TAAAGACCTCTATGCCAACAC	GATAGAGCCACCAATCCAC	166
MYH6^c^ (alpha myosin heavy chain 6)	TTCCGCAAGGTGCAGCACGAG	TCCTCATCGTGCATTTTCTGCTTGG	125
MYH7^c^ (beta myosin heavy chain 7)	TTCCGCAAGGTGCAGCACGAG	TACTCTTCATTCAGGCCCTTGGCGC	122
Cx-43^b^ (connexin 43)	GTTCTATGTGATGAGGAAGG	ACTTCTTGATTTCAATCTGC	116

a, b Primers have been described earlier (24), (9), respectively;

c Primers have been made with Primer-BLAST of NCBI.

### Quantitative RT-PCR

Quantitative RT-PCR was performed according to the standard protocols on an Abi Prism 7300 instrument (Applied Biosystems, Foster City, CA, USA). Total RNA was isolated as described above. Complementary DNA was synthesized from 200 ng of total RNA in a total volume of 20 µl as described above. The PCR reaction consisted of 1 µl cDNA, 7.5 µl of 2 × Power SYBR green PCR mastermix (Applied Biosystems, Foster City, CA, USA), 5.3 µl sterile water, and 400 nM of each primer, which were for the same markers as those in RT-PCR (Table 2). Each experiment group was analyzed separately and at least two replicates between both the stimulated and control groups were analyzed as triplicates. Cτ values were determined for every reaction, and the relative quantification was defined with the 2^-ΔΔCτ^ method (18). The data were normalized to the expression of the housekeeping gene Beta actin (β-actin), and the unstimulated sample for each test series served as the calibrator. Statistical significance for each experiment group was determined with the t-test.

### Atomic force microscopy

Atomic force microscope (AFM) (XE-100, Park Systems, Korea) with ACTA-50 probe was used in studying the surface of the MEA dish. AFM images of an empty MEA surface were obtained by moving the probe on the sample surface across the measurement area in one direction for each image line in order to create a topographical map of the sample (7).

## RESULTS

### Stimulator capabilities

The designed system, consisting of the MEA container, stimulation electronics, and the controlling software on a laptop computer, fulfilled the requirements set for the device. To our knowledge, the resulting system is among the first systems to enable the use of the MEA platform in long-term electrical stimulation with homogeneous fields.

The difference of medium pH levels in control and stimulated samples after long term stimulation was 0.10% suggesting stimulation had no effect on pH levels. There were either seen no degradation of the electrodes or any possible gas formation during the different stimulation protocols.

### Cardiomyocyte function in a short-term pacing experiment

The effect of short-term stimulation on cell beating was characterized by a pacing experiment in which different stimulation parameters were applied and the pacing of the cells observed visually with a microscope. Initially, the beating rate was about 60 per minute. As the stimulation stopped, beating returned to its original, spontaneous frequency, so the cells did not remain in the paced rhythm after stimulation (data not shown). Visually, the pulse duration or pulse form had no effect on cell beating. When the electrical field was 5 V/cm, the beating was stronger than with the smaller 2.5 V/cm field.

### Cell morphology

**Figure 3:**
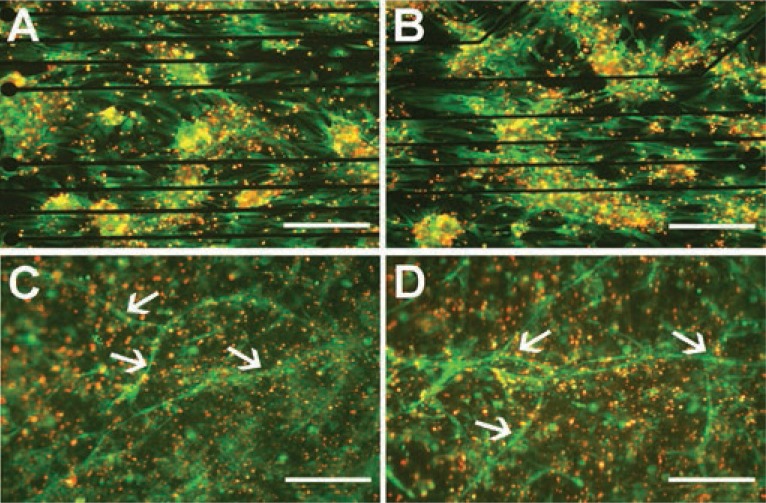
Cell viability. In addition to cell viability, the orientation of NRCs on gelatin coating parallel to the electrode lines of the MEA chambers is evident in the live/dead stainings (live cells green, dead cells red) in both control **(A)** and stimulated **(B)** samples. The tube formation of cells on collagen gel in experiment IV is clearly evident from the live/dead stainings in both control (**C**) and stimulated **(D)** samples. Arrows denote tube-like structures, which were observed in both stimulated and control samples. The direction of the electrical field is horizontal in the pictures. Scale bars are 200 µm.

**Figure 4:**
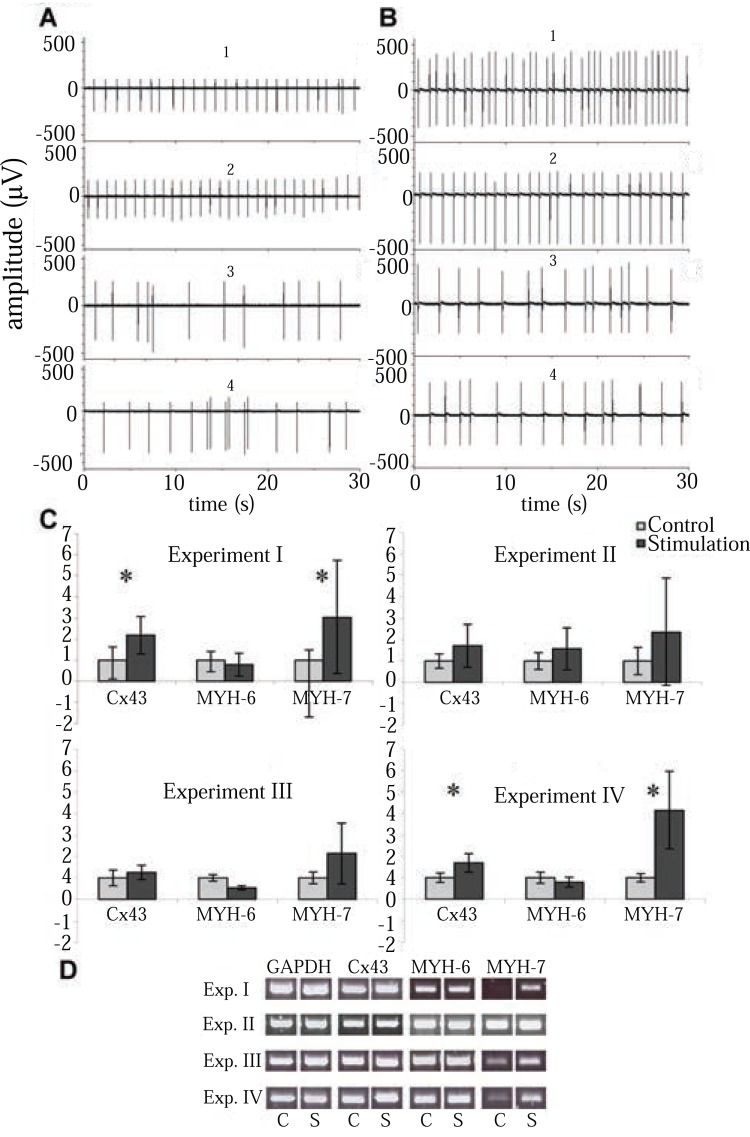
Functional properties of long-term stimulated NRCs. Electrical activity of control NRCs **(A)** and stimulated samples **(B)** in experiment II before stimulation (1) and after 5 hours (2), 24 hours (3), and 48 hours (4) of stimulation. **(C)** Cx-43, MYH-6, and MYH-7 expressions from each experiment determined by qPCR at the end of culturing. Statistical significance is marked with (*p<0.05). Number of control vs stimulated samples analyzed as triplicates: Exp. I n=2 vs n=3, Exp. II n=3 vs n=4, Exp. III n=2 vs n=2, Exp. IV n=3 vs n=3. Error bars are SD. **(D)** Gene expressions from each experiment determined by RT-PCR at the end of culturing. S, electrically stimulated samples; C, control samples.

In the long-term stimulation, cells in experiments I, II, and III (Table 1) were electrically stimulated on gelatin-coated MEA chambers with different parameters (Figure 2). Different parameters based on earlier publications (3, 9, 24) were tested to try to find the optimal parameters for NRC stimulation. The morphology of NRCs was the same during stimulation, and both stimulated and control cells exhibited similar morphological features throughout the experimental period. The cells were not elongated or oriented parallel to the electrical field. Instead, the orientation of the cells was seen parallel to the MEA electrode wires in both the control and stimulation samples (Figures 3A and 3B). An equal number of cells were plated on each well, and the number of cells decreased similarly during the culture in both the control and stimulated samples. The time in culture also affected the beating frequency, which was evident both microscopically and in MEA measurements as a decrease in the beating rate (Figures 4A and 4B).

In experiment series IV, CMs were cultured on collagen gel (Figures 3C and 3D). The cells contracted nicely and were well spread over the surface of the gel. After three days of stimulation, cell orientation appeared unaltered. Microscope analysis revealed that cells in the control and stimulation samples were similarly shaped. In both the control and stimulated samples, long tube-like structures (Figure 3C and 3D) were observed on the collagen gel in addition to cardiac cells.

### Electrophysiology

We monitored the electrical activity of both stimulated and control NRCs with MEA. The spontaneous beating rate varied between 0.5 and 2 Hz, and was usually 1.0 Hz (Figures 4A and 4B). After stimulation, the cells either stopped beating or the beating rate was more irregular than before stimulation; this was also evident in the controls after experiment. In experiment II, the signals were measured before stimulation and 5, 24, and 48 hours after stimulation. The control samples were measured at the same time points. The beating rate decreased both in stimulated and control groups (Figure 4A and B), but less radically than in experiments I, III and IV. The beating was more irregular in the end of the experiment than in the beginning (Figure 4A and 4B).

MEA measurements could not be carried out on cells on collagen gel due to the thickness of the gel, so the beating was observed only visually with a microscope. Before stimulation, the beating rate of the cells on the gel was regular, contractions were clearly observed, and the gel adjusted to the beating. Although the size of the beating area differed between replicates, overall the beating was the same when compared to the samples on gelatin coating. After stimulation, beating areas were fewer in number and smaller in size, and the beating frequency in both sample groups decreased.

### Cell viability

Cell viability was determined after each experimental series. Live/dead staining indicated that there were no differences in cell viability between the stimulated and control samples (Figures 3A, 3B, 3C, and 3D) and roughly 50 % of the cells were alive in both conditions. In addition, cell alignment and elongation was more distinguishable with the help of live/dead staining, but we observed no changes due to stimulation. Instead, the orientation of the cells on gelatin coating was parallel to the MEA chamber electrode wires in both the control and stimulation samples (Figures 3A and 3B). The orientation was very clear in experiment II, and cell alignment was precisely paralleled the direction of the electrode wires.

With the help of live/dead staining, we observed the tube-like structures from the collagen gel samples. Tubes were present in every collagen gel sample (Figures 3C and 3D) and showed no orientation in any specific direction.

### Gene expression

According to the RT-PCR results, the MYH-7 mRNA level was higher in the 3-day stimulation experiments (I, III, and IV) than in the control samples. Cx-43 expression levels were also slightly elevated in these stimulated samples, but in other genes no differences were evident (Figure 4D).

To ensure the difference in gene expression levels, quantitative RT-PCR was carried out for the same samples as used in RT-PCR. Stimulation increased MYH-7 and Cx-43 expression levels in experiments I and IV (*p*<0.05) when compared to controls (Figure 4C). In experiments II and III, MYH-7 and Cx-43 expression levels had a trend towards higher expression, but it did not reach statistical significance. No significant difference in MYH-6 expression between the control and stimulated samples was observed in any experiment.

### Protein expression

**Figure 5:**
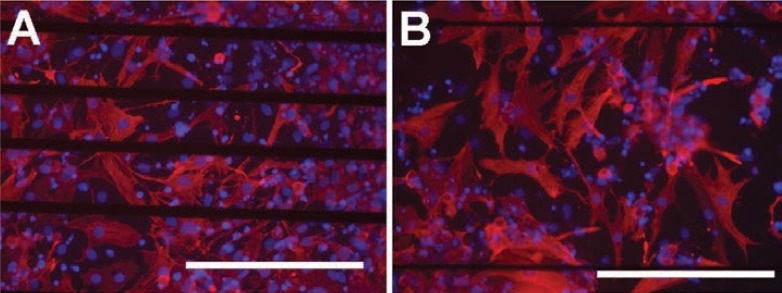
Expression of troponin T (red) in control (**A**) and in stimulated (**B**) samples; blue indicates cell nuclei (scale bars 200 µm).

Immunocytochemistry served to identify CM structures. The stimulated cells stained positively with troponin T or I similarly to the controls (Figure 5). The stimulated CMs revealed no visible changes in sarcomeric structure.

In experiment IV, narrow and long tube-like structures formed during culture on collagen gel. The tubes were analyzed with antibodies against Vimentin (mesenchymal origin: fibroblasts, endothelial, smooth muscle), α-AS (myotubes in skeletal and cardiac muscles), α-SMA (smooth muscle), VWf1 (endothelial), VWf2 (endothelial), Pax-6 (neural) and MAP-2 (neural), however, no positive staining with any antibodies used was seen (13). Since the origin of tubes were not neural, vascular or muscle-derived the nature of the tubes remains unknown.

### Surface measurement using AFM

**Figure 6:**
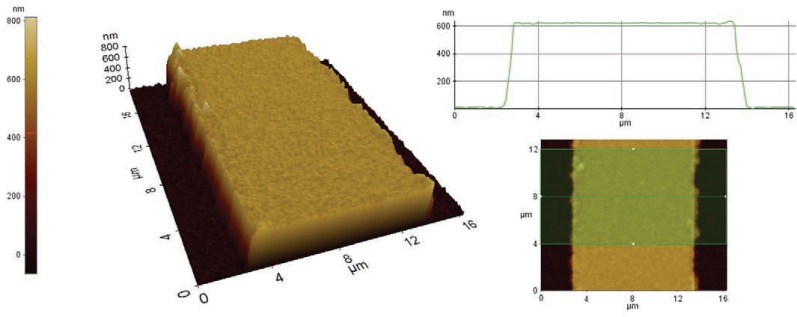
An AFM surface scan of the MEA electrode wiring. The scan shows the height of the electrode wirings to be approximately 600 nm. The height was determined by averaging the height profile from an 8-µm-long wiring track.

To estimate the effect of surface abrasions on the orientation of cells, AFM served to measure the height of the electrode wires on an MEA dish. The results revealed that the electrode wires on the MEA had a height of 600 nm (Figure 6).

## DISCUSSION

A novel platform for the electrical stimulation of cells was designed in this study. When compared with other existing systems for cell culture stimulation, the designed system provides several possibilities which to our knowledge can not all be found in other stimulation platforms: the possibility to stimulate several cell cultures, homogeneous long term electrical field stimulation on MEAs and programmable stimulation platform. In some earlier studies MEAs have been used (8, 16), which have the capability of localized electrical stimuli. However in studies with goal of creating a uniform field, carbon rod electrodes have been more widely used (3, 12, 24) together with commercial cell containers like plates, wells and dishes (3, 12, 24). In waveform creation commercial stimulators are used in several studies (3, 11, 24).

The main objective was to examine whether electrical field stimulation affects synchronous cell contractions and cell alignment, the maturation or functional assembly of CMs. In the present study, stimulation was initiated on either the second or third day after plating. This arrangement was based on previous findings (24), which indicate that if applied on the first day in vitro, electrical stimulation yields poor contractile behavior. In contrast, if stimulation was applied on day five, fewer contractile proteins were found in the cells (24). This timing also ensured that the cells had enough time to recover from the cell isolation procedure and to attach properly to the MEA chambers prior to stimulation. In the present study both monophasic and symmetric biphasic square pulses were used. A previous study has reported that the use of biphasic pulses in applications of electrical stimulation maintains biocompatibility better by avoiding tissue injury or electrode damage (25). Biphasic pulses minimize the damage caused by electrodes as a result of the redox reaction on the electrodes, which leads to tissue damage due to pH and metal salts released in the process (25). Biphasic pulses have also been reported to enhance the positive effects of electrical stimulation with regard to functional and structural properties (9). However, we saw no differences between biphasic and monophasic pulses in our set-up.

In the pacing experiments the stimulator served to pace NRCs and in the absence of stimulation the beating returned back to spontaneous rhythm. When the field strength was 2.5 V/cm, the cells failed to contract as uniformly as with the higher 5 V/cm fields, thus demonstrating the efficacy of the system. Previous studies have shown that the higher the field strength, the more likely the cell will reach the depolarization threshold and contract in response to the stimulus (3).

The benefit of the new stimulation system is its potential to stimulate cells in MEA chambers and measure the electrical activity of cells both before and after stimulation. In the long-term experiments, the beating rate decreased in all samples. The detachment of the cells affected negatively on the spontaneous beating rate which might be due to the loss of the cell-to-cell contacts necessary for signal induction. CMs isolated from the heart and cultured *in vitro* are known to dedifferentiate and eventually stop beating (21). They also lose many of their surface channels and receptors (2), myofilament equipment, and acquire a round shape (35). All these factors may lower the beating rate, which was evident in the MEA measurements. Previous studies indicate that the number of spontaneously beating cells decreased to 8% after eight days *in vitro* and no spontaneous beating was observed in the stimulated samples, but the beating could be triggered by external stimulus (26).

Live/dead staining revealed that there were no significant differences in cell viability between the stimulated and control samples. In earlier studies, the cells were shown to elongate, couple, and orientate parallel to the electrical field (3, 11, 24) using either a direct (3) or an alternating current field (11). In the present study no such morphological changes in the cells were observed. However, no changes in cell morphology during electrical stimulation has also reported earlier (26). Collagen gel could also provide a more elastic surface to enable cell orientation. However, we found no improvement in the stimulation results, but the cells spread nicely and migrated within the gel, and the gel allowed the cell beating.

In the present study, cells were oriented parallel to the MEA chamber electrode wires in both the control and stimulation samples, so the topography of the cell culture surface was a determinant of cell orientation. The AFM results showed that the electrode wires on the MEA have a height of 600 nm. Previous studies have shown that topographical cues of a height of several hundred nanometers are sufficient to induce cell orientation (3, 4, 15). Au *et al*. used topographical cues from 140 to 700 nm of height (3) as well as microgrooves of 400 nm (4) which were all capable of inducing cellular orientation. Kim *et al*. used nanoscale cues from 200 to 500 nm in height, and these affected the structure and function of cardiac tissue constructs (15). Our findings support those earlier observations that the surface topography is a stronger factor than the electrical field with regards to cell orientation; once cells have orientated towards topographical shapes, they cannot re-orient with electrical stimulation (3, 4). We can therefore conclude that when aiming at cell orientation, it is more worthwhile to model and improve the topography of the cell substrate than to use electrical field stimulation.

Previous studies have shown that electrical field stimulation influences CM functionality (9, 24, 26). Cx-43 molecules form gap junctions, which are intercellular channels located in the intercalated disc and provide both electrical and metabolic coupling for CMs (33). Cx-43 is expressed in virtually all myocytes of the atrial- and ventricular mammalian myocardium regardless of the stage of development (33). According to the qPCR analysis in our study, the expression level of Cx-43 was significantly higher in two stimulation experiments when compared to the controls. This is in line with earlier studies where the expression of Cx-43 increased on both the gene and protein levels due to electrical field stimulation (3, 9, 24). MHC is the major contractile protein of cardiac muscle cells and the primary determinant of the efficiency of muscle contraction (32). MHC genes exist in cardiac cells in two isoforms: α-MHC encoded by the MYH-6 gene, which is upregulated during adult cardiac development, and β-MHC by the MYH-7 gene, which is upregulated during fetal cardiac development (22). In this study, the gene expression level of MYH-7 increased after three days of stimulation, which suggests an increase in the fetal type of cardiac cells due to electrical stimulation. Stimulating the CMs for two days instead of three showed no differences in MYH-7 expression, which may indicate that stimulation should last at least three days to ensure perceptivity of the effects of stimulation. These results are in line with previous studies where the expression of sarcomeric proteins MYH-6 and MYH-7 increased due to electrical stimulation and its duration. However, in the earlier reports the ratio of these two isoforms has changed indicating maturation of the CMs (24). We did not observe this in our study. Other studies have shown that the mRNA level of MYH-7 in CMs derived from hESCs is substantially higher in ventricular tissue (1) which has also been observed in rat hearts (29). This could indicate that most isolated and cultured NRCs were ventricular, and therefore only MYH-7 expression increased. One reason for the increase in MYH-7 expression could be the mechanical stress, in this case due to electrical stimulation, which in earlier studies induced a shift from α-MHC toward β-MHC composition in mice hearts (17). This shift could reduce contractile efficiency, and therefore be one reason for the lower beating rate observed in this study.

As a conclusion our data suggest that surface topography was a stronger determinant of CM orientation than electrical field stimulation; consequently, the micro architecture of the materials should be examined carefully when cell orientation is desired. Electrical stimulation and its duration seem to affect gene expression profile, and this stimulation system may prove useful in the future, for example, to enhance cardiac differentiation of stem cells. Our stimulation platform proved capable of pacing cultured cells, and this property can be exploited in various cardiac-related studies requiring the electromechanical stimulation of cardiac cells. However, we did not detect any long-term effects of electrical stimulation on the electrophysiological behavior of the cells even though stimulation increased Cx-43 expression. In the future, it would be important to optimize the stimulation parameters to improve the maturation process and to understand the effect of these parameters at the cellular level. It would also be important to understand better the mechanism of how electrical stimulation influences cell differentiation and maturation and to discover effective ways to quantify this phenomenon at the cellular level.
